# Healthy Food Benefit Programs, Fruit and Vegetable Consumption, and Food Security

**DOI:** 10.1001/jamanetworkopen.2025.27601

**Published:** 2025-08-19

**Authors:** Melissa A. Knox, Jamie Wallace, Barbara Baquero, KeliAnne Hara-Hubbard, Jessica Jones-Smith

**Affiliations:** 1 Department of Economics, University of Washington, Seattle; 2 Department of Health Systems and Population Health, University of Washington, Seattle

## Abstract

**Importance:**

Diet quality, including fruit and vegetable intake, is a determinant of health, yet fresh fruits and vegetables are inaccessible to many lower-income and marginalized households. Healthy food benefit programs may reduce health disparities by increasing fruit and vegetable consumption.

**Objectives:**

To estimate the outcomes of a $40 per month healthy food benefit program on food security and diet.

**Design, Setting, and Participants:**

Cohort study with randomly assigned treatment groups composed of participants seeking enrollment in Fresh Bucks, a healthy food benefit program for lower-income populations in Seattle, Washington. Participants were randomly assigned to enrollment in the program and to a waiting list. Outcomes were examined at baseline (October 2021) and follow-up (June 2022).

**Exposure:**

Two exposure groups were examined. First, random assignment to newly receive program benefits for 6 months vs assignment to a waiting list. Second, random assignment to be disenrolled from program benefits vs continued enrollment for 6 months.

**Main Outcomes and Measures:**

A modified version of the Behavioral Risk Factor Surveillance System Fruit and Vegetable Screening Questionnaire was used to calculate daily frequency of fruit and vegetable consumption as a continuous outcome and a binary indicator of consuming less than 3 vs 3 or more times per day. Food security was measured using the 2-item Hunger Vital Signs screener.

**Results:**

A total of 1973 participants were included, with 757 who received the program and 1216 who were disenrolled (1339 [68%] aged ≥60 years; 1007 [51%] Asian; 209 [11%] Black; 523 [27%] White). The program was associated with a 5.5 percentage point (pp) (95% CI, 0.05-10.91 pp) increase in food security and a 7.5 pp (95% CI, 0.39-14.52 pp) increase in the proportion of participants consuming fruit and vegetables 3 or more times per day. Continuous fruit and vegetable consumption changes were null; however, differential outcomes by income, language, and race and ethnicity were found. Examining these same outcomes in a population assigned to disenrollment, lower fruit and vegetable intake and food security were found vs those who remained in the program.

**Conclusions and Relevance:**

This cohort study of a healthy food benefit program found reduced food insecurity and improved fruit and vegetable consumption in a lower-income population randomly assigned to program enrollment.

## Key Points

**Question:**

Is a healthy food benefit program for low-income households associated with fruit and vegetable intake and food security?

**Findings:**

This cohort study with 1973 participants found the healthy food benefit program was associated with a 5.5 percentage point–increase in food security and a 7.5 percentage point–increase in the proportion of participants consuming fruits and vegetables 3 or more times per day in enrolled vs wait-listed households.

**Meaning:**

These findings suggest healthy food benefit programs could improve fruit and vegetable intake and food security among low-income populations.

## Teaser

This cohort study evaluates the outcomes of a monthly healthy food benefit program on food security and diet in Seattle.

## Introduction

Diet quality, including fruit and vegetable intake, impacts risk for premature disability and death from cardiometabolic disease, cancer, and other causes.^1,2,3^ But fresh fruits and vegetables tend to be more expensive than processed foods and less available in lower-income neighborhoods.^4,5^ Food insecurity, or the limited or uncertain availability of nutritionally adequate and safe foods, is also linked to lower incomes and is associated with poor nutrient intake, diabetes, and hypertension.^6,7^ Furthermore, other social factors by which people are marginalized, such as race, ethnicity, and nativity, are also associated with higher prevalence of food insecurity and lower intake of fruits and vegetables.^8,9,10^ Accordingly, diet may be a pathway through which social determinants create health inequities.

Programs that make fruits and vegetables more accessible and improve food security for lower-income families have potential to reduce health disparities and improve population health. Both public and private insurers have shown increased interest and investment in food is medicine (FIM) programs, including produce prescriptions and medically tailored meals that provide free, healthy foods to patients. Before the popularity of FIM, federal Food Insecurity Nutrition Incentive grants funded state- and local-level nutrition incentive programs to increase access to healthy foods and improve food security for lower-income populations. These programs matched a portion of household spending on fruits and vegetables, were limited to households receiving Supplemental Nutrition Assistance Program (SNAP), and were often only redeemable at farmers markets.^11,12^

Much of the existing literature evaluating FIM or the older government-funded programs is hampered by noncausal research designs.^13^ One study of 9 produce prescription plans found reduced food insecurity and improved diet, but the evaluation lacked a comparison group.^14^ A few rigorous evaluations employ randomization, but findings are inconsistent. One randomized evaluation of medically tailored meals found that treatment improved a diet quality score, but not fruit and vegetable consumption or health.^15^ Similarly, 2 randomized evaluations of government-funded matched spending programs found that household purchases of fruits and vegetables increased, but only 1 of these found dietary changes.^16,17^

This study contributes to the literature on the outcomes of healthy food benefit programs by using a rigorous study design to evaluate a fruit and vegetable benefit program available to lower-income households in Seattle, Washington. We leveraged randomized program assignment to assess the outcomes of both gaining access to the program for 6 months and of losing access to the program for 6 months after over 1 year of receipt. The program has several innovative characteristics that have not been previously examined in the literature. These include no required participant spending match and sustainable financing through a beverage tax, focused enrollment of households not eligible for other nutrition assistance programs and no requirement for SNAP participation, and the ability to redeem benefits at a large chain food retailer and smaller local stores located in lower-income neighborhoods.

## Methods

The University of Washington institutional review board determined that this study was not research, and informed consent was not required because surveys were collected by the City of Seattle for program evaluation purposes. This study followed Strengthening the Reporting of Observational Studies in Epidemiology (STROBE) reporting guidelines.

### Program Background

Seattle’s Fresh Bucks is a $40 per month benefit that can be used to purchase fresh fruits and vegetables at 17 Seattle locations of a large grocery chain and all 15 Seattle farmer’s markets, or to purchase fresh, frozen, canned, and dried fruits and vegetables at 6 independently owned grocery stores. The benefit is distributed via app or electronic benefits card and can be used in any denomination across multiple shopping trips within the benefit month. Available to Seattle residents with incomes less than 80% of the area median income ($90 500, or 370% of the federal poverty level (FPL) for a family of 4 in 2020) and not requiring SNAP eligibility, the program began in 2020 and continuously enrolled its first beneficiaries through the end of 2021. There are 2 modes of enrollment: an online public enrollment application, and enrollment via outreach from community-based organizations.

### Study Sample and Data

In October 2021, both new applicants and continuing 2020 to 2021 beneficiaries were required to complete the program application survey to (re)apply to receive benefits in 2022. Since the 6900 total applications exceeded program funding, 4200 were chosen for enrollment via random draw, with the remaining 2700 placed on a waiting list (eFigure 1 and eTable 1 in Supplement 1). In July 2022, the City of Seattle mailed a follow-up survey to all 6900 applicants, both beneficiaries and waiting list members. Our study sample consists of the 1973 who completed and returned surveys. Data for this study come from responses to both rounds of the administered survey as well as program administrative data containing the results of the random enrollment draw.

From the sample who returned surveys, we constructed 2 treatment groups for our study. Intervention group 1 (IG1) studies the outcomes of new program enrollment by comparing new applicants treated via random assignment to program enrollment with those assigned to the waiting list. Intervention group 2 (IG2) studies the outcome of losing the program benefit by comparing returning applicants treated via random assignment to the waiting list with those assigned to continue receiving benefits (eFigure 1 in Supplement 1).

### Outcomes

#### Food Security

Food security was measured via the validated 2-item Hunger Vital Signs food security screener. Response options were: often true, sometimes true, and never true. An answer of sometimes or often true to either of these 2 questions was treated as an indicator of household food insecurity in the past 12 months.

#### Daily Fruit and Vegetable Consumption

Fruit and vegetable consumption was measured with a modified version of the Behavioral Risk Factor Surveillance System Fruit and Vegetable Questionnaire, excluding 100% fruit juice.^18^ Five categories of fruits and vegetables were queried: fresh, frozen, or canned fruit of any type not including juice; leafy greens or lettuce salad; fried potatoes; nonfried potatoes; and all other vegetables. We converted all responses into frequencies per day.

#### Fruit and Vegetable Intake 3 or More Times Per Day

We chose the threshold of consuming fruit and vegetables less than 3 total times per day to indicate low consumption of fruits and vegetables.^19^ We reverse coded to match the direction of other outcomes.

We estimated power for our study to detect changes in fruit and vegetable consumption and food insecurity based on a 20% survey response rate in July 2022 (638 participants in IG1 and 760 participants in IG2). Assuming baseline fruit and vegetable consumption of 1.5 times per day and food insecurity of 85%, we used a 2-sided test with α = .05 to determine that we had power of 0.80 to detect a change in fruit and vegetable consumption of 0.29 times per day, and an 8 percentage point (pp) change in food insecurity.

### Covariates

While treatment was randomly assigned, we a priori chose to include covariates associated with the outcomes to increase precision. We used the least absolute shrinkage and selection operator (LASSO) to identify which covariates were most associated with each pretreatment outcome in a testing dataset (eMethods in Supplement 1). We included the following covariates and categories in all models: presence of children under 18 in household (yes or no), household income (>200% or ≤200% of FPL for household size), self-reported race and ethnicity (non-Hispanic African American or Black, Non-Hispanic Asian, Non-Hispanic White, another [includes American Indian or Alaska Native; Hispanic, Latino, or Spanish; Middle Eastern or North African; mixed race; Native Hawaiian or Pacific Islander; or prefer to self-describe], and missing [includes prefer not to answer]), age (above or below 60 years), preferred survey language (English, Chinese, Vietnamese, another [includes Amharic, Arabic, Khmer, Korean, Lao, Oromo, prefer to self-describe, Russian, Somali, Spanish, Tagalog, and Tigrinya], and missing [includes prefer not to answer]), and preferred retailer that accepts the program (indicator variable for households naming a retailer that accepts the program). We included household size as a normalized continuous variable.

### Effect Measure Modifiers

Consistent with the theory that social and spatial barriers affect food access,^20^ we hypothesized that treatment outcomes could be affected by language barriers, the cultural appropriateness of retailers in the program, and neighborhood food environment (eFigure 2 in Supplement 1). Thus, we examined treatment response heterogeneity by recipients’ income expressed as a percentage of the FPL, race and ethnicity, preferred language, and baseline food security status.

### Statistical Analysis

We estimated means and frequency distributions of demographic characteristics by intervention group and treatment or control group. We tested for statistically significant differences in demographic characteristics across treatment and control status within each intervention group using *t* tests for continuous variables and χ^2^ tests for categorical variables. We tested for selection into the analysis sample and discuss the generalizability of our results to all 2022 program applicants in eTable 2 in Supplement 1.

We estimated treatment outcomes separately for IG1 and IG2 using a linear regression of the posttreatment outcome (or linear probability model for binary outcomes) on 1 of the following treatment indicators: (1) random assignment to newly receive program benefits (IG1), or (2) random assignment to lose access to program benefits (IG2). Our analysis sample consisted of treated individuals and those randomized into a control group, and treatment outcomes were measured relative to control outcomes. All regression models controlled for baseline values of the outcome and all covariates described previously, following the analysis of covariance specification. This specification was chosen over a fixed effects or unadjusted comparison to increase power and precision (eMethods in Supplement 1).^21,22,23^ Heteroskedasticity-robust SEs and statistical significance at 1%, 5%, and 10% are reported in tables.

To assess heterogeneous treatment outcomes, we added an interaction between the treatment indicator and each effect measure modifier described previously. Due to power limitations, we only examined heterogeneous treatment outcomes for continuous fruit and vegetable consumption levels. We preregistered our analytic plan at Open Science Framework registries.^24^

We assessed whether our primary results were sensitive to using indicator variables for missing responses vs using a complete case analysis or to the inclusion or exclusion of potential outliers. In secondary analyses, we explored treatment outcomes on daily reported consumption for fruits and vegetables separately. Analyses were completed in Stata version 18.0 (StataCorp LLC).

## Results

### Descriptive Statistics

A total of 1973 participants are included in the analysis sample (1339 [68%] aged ≥60 years; 1007 [51%] Asian; 209 [11%] Black; 523 [27%] White), with 757 in IG1 and 1216 in IG2. Baseline characteristics of the full sample and by intervention group are displayed in Table 1. Approximately 80% of the sample lived in a household with 1 or 2 residents, had no children, and had a household income 200% of the FPL or less. The majority of the sample preferred either the English or Chinese languages. Thirty-one percent were aged 18 to 59 years, while 68% of the sample was aged 60 years or older. We found no statistically significant differences between the treated and control groups within intervention group, supporting the assumption that unobserved characteristics are also balanced across treatment status.

**Table 1.** Key Sample Characteristics by Treatment Group

Characteristic	Participants, No. (%)				
	Overall (N = 1973)	Gained program benefits		Lost program benefits	
		Enrolled (n = 430)	Wait-listed (n = 327)	Dropped (n = 517)	Continuous (n = 699)
Survey response rate	28.5	21.1	24.7	37.4	32.0
Children in the household					
No	1625 (82)	344 (80)	270 (83)	420 (81)	591 (85)
Yes	310 (16)	77 (18)	50 (15)	86 (17)	97 (14)
Prefer not to answer	38 (2.0)	9 (2.1)	7 (2.1)	11 (2.1)	11 (1.6)
Household income					
≤200% FPL	1674 (85)	336 (78)	263 (80)	458 (89)	617 (88)
>200% FPL	299 (15)	94 (22)	64 (20)	59 (11)	82 (12)
Household size					
1	1147 (58)	236 (55)	197 (60)	298 (58)	416 (60)
2	495 (25)	111 (26)	73 (22)	129 (25)	182 (26)
3	141 (7.0)	35 (8.1)	24 (7.3)	38 (7.4)	44 (6.3)
4	109 (6.0)	27 (6.3)	19 (5.8)	33 (6.4)	30 (4.3)
≥5	81 (4.0)	21 (4.9)	14 (4.3)	19 (3.7)	27 (3.9)
Race and ethnicity^a^					
Asian	1007 (51)	208 (48)	170 (52)	260 (50)	369 (53)
Black	209 (11)	55 (13)	36 (11)	51 (10)	67 (9.6)
White	523 (27)	111 (26)	82 (25)	143 (28)	187 (27)
Another race or ethnicity	147 (7.0)	34 (7.9)	22 (6.7)	43 (8.3)	48 (6.9)
Missing	87 (4.0)	22 (5.1)	17 (5.2)	20 (3.9)	28 (4.0)
Preferred language^b^					
English	1046 (53)	233 (54)	179 (55)	278 (54)	356 (51)
Chinese	572 (29)	113 (26)	83 (25)	156 (30)	220 (32)
Vietnamese	204 (10)	41 (9.5)	32 (9.8)	54 (10)	77 (11)
Another language	139 (7.0)	39 (9.1)	31 (9.5)	26 (5.0)	43 (6.2)
Missing	12 (0.61)	4 (0.93)	2 (0.61)	3 (0.58)	3 (0.43)
Age, y					
18-59	620 (31)	168 (39)	126 (39)	142 (28)	184 (26)
≥60	1339 (68)	259 (60)	197 (60)	372 (72)	511 (73)
Missing	14 (0.71)	3 (0.69)	4 (1.2)	3 (0.58)	4 (0.57)

Abbreviation: FPL, federal poverty level.

^a^ Another race and ethnicity includes American Indian or Alaska Native, Hispanic, Middle Eastern or North African, mixed race, Native Hawaiian or Pacific Islander, or prefer to self-describe.

^b^ Another language includes Amharic, Arabic, Khmer, Korean, Lao, Oromo, prefer to self-describe, Russian, Somali, Spanish, Tagalog, and Tigrinya.

The eMethods in Supplement 1 shows demographic characteristics for the full population of October 2021 applicants and discusses potential selection into the analysis sample. Except for income, all demographic characteristics were balanced across treatment status within both intervention groups in the full population (eTable 1 in Supplement 1). Some groups (older, Vietnamese speaking and those on the waiting list in both groups, higher-income in intervention group 1, and White in intervention group 2) were more likely to respond to the survey and are consequently overrepresented in the analysis sample (eTable 2 in Supplement 1). eTable 3 and eTable 4 show missingness in baseline and follow-up survey responses, respectively. The overall survey response rate was 28.5%. Unadjusted baseline levels of outcome measures were similar for all outcomes within intervention groups, while change over time appeared to differ by treatment status (Table 2).

**Table 2.** Baseline and End Line Outcomes by Treatment Group

Outcome	Participants, No. (%)			
	Gained program benefits		Lost program benefits	
	Enrolled (n = 430)	Wait-listed (n = 327)	Dropped (n = 517)	Continuous (n = 699)
Binary food security^a^				
Baseline	67 (16)	52 (16)	89 (17)	123 (18)
Endline	105 (25)	67 (21)	110 (21)	182 (26)
Continuous fruit and vegetable intake/d, mean (SD)^b^				
Baseline	1.95 (1.66)	2.00 (1.67)	2.28 (1.64)	2.33 (1.64)
Endline	3.11 (1.76)	2.93 (1.96)	2.80 (1.89)	3.20 (1.86)
Binary fruit and vegetable intake ≥3 times/d^c^				
Baseline	80 (19)	65 (20)	138 (27)	199 (28)
Endline	205 (48)	133 (41)	203 (39)	330 (47)

^a^ Food security is a binary outcome based on a 2-question food insecurity screener.

^b^ Fruit and vegetable consumption is a continuous outcome calculated based on a modified version of the Behavioral Risk Factor Surveillance System Fruit and Vegetable Screening Questionnaire.

^c^ Fruit and vegetable intake more than 3 times per day is a binary outcome that is 1 when fruit and vegetable intake per day is 3 or more.

### Average Treatment Outcomes

#### Outcomes of Gaining Program Benefits

Relative to placement on a waiting list, gaining program benefits (Table 3) was associated with increased food security by 5.48 pp (95% CI, 0.05-10.91 pp), or 31% of baseline. Gaining program benefits was also associated with an increase in the rate of consuming fruits and vegetables more than 3 times per day by 7.45 pp (95% CI, 0.39-14.52 pp), or 37%. Changes in continuous fruit and vegetable intake were positive, but not statistically significant.

**Table 3.** Average Treatment Outcome for New Enrollment and Dropped Enrollment^a^

Treatment outcome	Gained program benefits (intervention group 1)		Lost program benefits (intervention group 2)	
	Coefficient (95% CI)	*P* value	Coefficient (95% CI)	*P* value
Food security prevalence	5.48 (0.05 to 10.91)^b^	.048	−4.97 (−9.34 to −0.59)^b^	.03
No.	755		1208	
Continuous fruit and vegetable consumption	0.22 (−0.05 to 0.49)	.11	−0.37 (−0.58 to −0.16)^c^	.001
No.	722		1183	
Fruit and vegetable consumption 3 times/d prevalence	7.45 (0.39 to 14.52)^b^	.04	−7.34 (−12.82 to −1.86)^c^	.01
No.	756		1216	

^a^ Linear regression with covariates: standardized household size, indicator for presence of children in the household, race and ethnicity categories, indicator for income more than 200% of the federal poverty level, language category, indicator for age 60 years or older, indicator for preferred retailer accepts program benefits. Baseline outcome also included as covariate. Regression results for food security and fruit and vegetable consumption 3 times per day exclude those with missing data at follow-up for food security or fruit and vegetable consumption, respectively. Regression results for continuous fruit and vegetable consumption exclude those with missing fruit and vegetable consumption at either baseline or follow-up.

^b^ *P* < .05.

^c^ *P* < .01.

#### Outcomes of Losing Program Benefits

Relative to continuing enrollment, losing program benefits and being placed on a waiting list (Table 3) was associated with reduced food security by 4.97 pp (95% CI, −9.34 to −0.59 pp), or 29% of baseline. Losing program benefits was also associated with eating fruits and vegetables 0.37 fewer times a day (95% CI, −0.58 to −0.16), or 16%, and a 7.34 pp decrease (95% CI, −12.82 to −1.86 pp) in consuming fruits and vegetables 3 or more times per day, or 26% .

### Treatment Outcome Heterogeneity

#### Outcomes of Gaining Program Benefits

**Figure 1.**
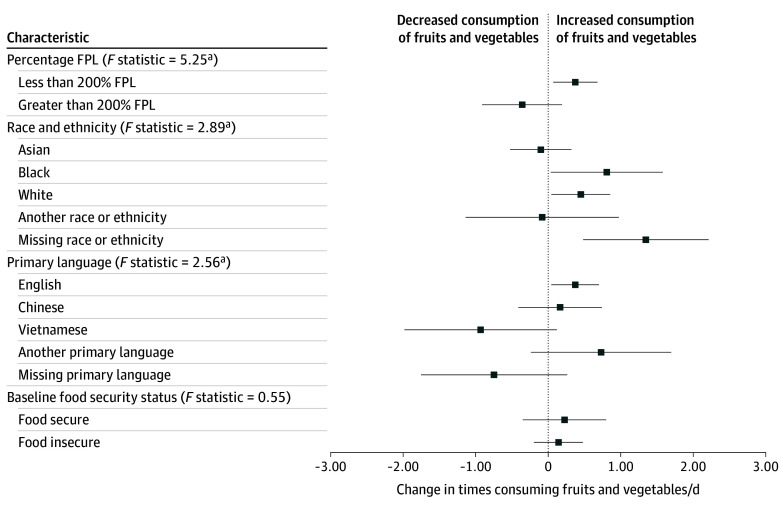
Treatment Outcome for New Enrollment by Income and Demographics Regression results shown in eTables 5 through 8 in Supplement 1. Race and ethnicity and language are self-reported. Another race or ethnicity includes American Indian or Alaska Native, Hispanic, Middle Eastern or North African, mixed race, Native Hawaiian or Pacific Islander, or prefer to self-describe. Another language includes Amharic, Arabic, Khmer, Korean, Lao, Oromo, prefer to self-describe, Russian, Somali, Spanish, Tagalog, and Tigrinya. FPL indicates federal povery level. ^a^P < .05.

The association of gaining program benefits with fruit and vegetable consumption was positive for people with income 200% FPL or lower (0.37; 95% CI, 0.07 to 0.68) and null for those with incomes over 200% FPL (Figure 1). There were also associations for White (0.45; 95% CI, 0.04 to 0.86) and Black (0.81; 95% CI, 0.04 to 1.58) individuals, and those with missing ethnicity information (1.35; 95% CI, 0.48 to 2.21), but there was no association for Asian individuals. Gaining access to the program was associated with increased fruit and vegetable consumption for English speakers (0.04; 95% CI, 0.04 to 0.70) but not for speakers of another language. However, the difference for Vietnamese speakers was negative and but was not statistically significant (−0.93; 95% CI, −1.98 to 0.12) (eTable 7 in Supplement 1). There was no outcome heterogeneity by baseline food security (eTable 8 in Supplement 1).

#### Outcomes of Losing Program Benefits

**Figure 2.**
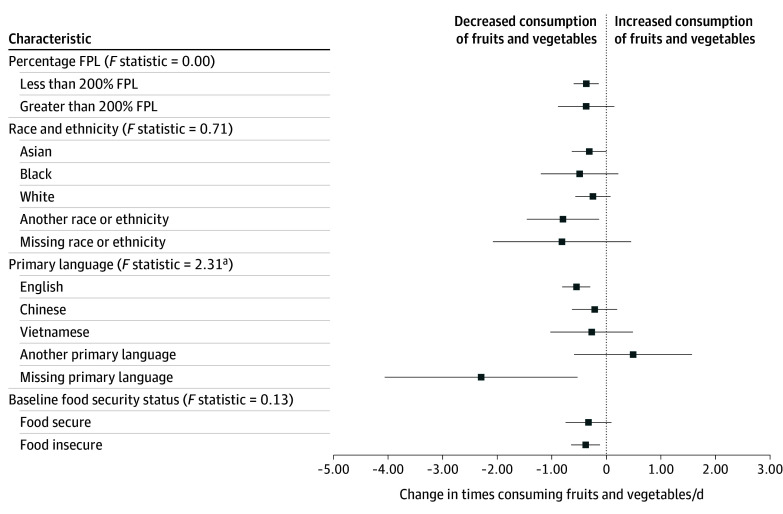
Treatment Outcome for Dropped Enrollment by Income and Demographics Regression results shown in eTables 5 through 8 in Supplement 1. Race and ethnicity and language are self-reported. Another race or ethnicity includes American Indian or Alaska Native, Hispanic, Middle Eastern or North African, mixed race, Native Hawaiian or Pacific Islander, or prefer to self-describe. Another language includes Amharic, Arabic, Khmer, Korean, Lao, Oromo, prefer to self-describe, Russian, Somali, Spanish, Tagalog, and Tigrinya. FPL indicates federal povery level. ^a^P < .01.

Figure 2 shows the association between losing program benefits for 6 months with fruit and vegetable consumption by the same characteristics as previously mentioned. There was no outcome heterogeneity by income, race and ethnicity, or baseline food security. However, there was some evidence for heterogeneity in effects for those with missing or another language (eTable 7 in Supplement 1).

### Sensitivity and Secondary Analysis

Results were substantively unchanged in the complete case analysis (eTable 9 in Supplement 1). In secondary analyses, gaining program benefits was associated with increased consumption of fruit of 0.20 times per day, and there was no change to vegetable consumption (eTable 11 in Supplement 1). Conversely, losing program benefits was associated with decreased fruit consumption of 0.15 times per day and vegetable consumption of 0.21 times per day.

## Discussion

This study evaluated the outcomes of a $40 per month voucher for fruits and vegetables by leveraging random program assignment to either receive the voucher or be placed on a waiting list. We found that access to the program was associated with improved fruit and vegetable consumption and food security in lower-income populations. Using the same design to study previously enrolled beneficiaries randomly assigned to no longer receive the benefit, we found strong evidence that dietary changes and food security did not persist once the benefit is lost. We found heterogeneous associations between receiving the program and continuous fruit and vegetable consumption by income level, racial identity, and language, but not by baseline food security status. The association between losing the program and fruit and vegetable consumption was homogeneous across most characteristics except language.

Our findings that new receipt of this healthy food benefit program was associated with improved food security and that loss of program benefits was associated with decreased food security are consistent with work that found that the odds of experiencing food insecurity decreased by one-third for recipients of a produce prescription program (from a baseline of 65%).^14^ However, that study lacks a comparison group or causal research design. Consequently, our study provides substantially more evidence than previous research that nutrition incentives can reduce food insecurity.

We also contribute to the limited store of evidence that healthy food benefit programs can positively impact fruit and vegetable intake. Our finding that the program increased the proportion of program participants consuming fruit and vegetables 3 or more times a day by 37% is consistent with one finding that a produce discount program increased fruit and vegetable intake by 26%.^16^ However, other randomized evaluations of similar programs find either no impact on diet^17^ or limited evidence from impact on a diet index score without a measurable change in the consumption of either fruits or vegetables.^15^

Examining continuous fruit and vegetable intake, we found that the outcomes of the program were positive but not statistically significant on average, but that this null outcome masks positive associations for some populations. Specifically, the program was associated with greater continuous fruit and vegetable intake for lower-income (vs higher-income) individuals, individuals who are White, Black, or missing race and ethnicity information (vs Asian or another race), and individuals who preferred to speak English (vs Vietnamese or missing language). One potential explanation for these results is that some groups use comparatively more of the benefit because they experience fewer language or cultural barriers to redeeming the benefit. Another is that some spend their benefit to reduce cash spending without changing overall produce purchases. These hypotheses deserve further investigation.

We found that losing access to a healthy food benefit program was negatively associated with fruit and vegetable consumption and food security overall. Previous studies^25,26^ have found similar results and suggest that without funds to purchase foods, dietary changes are not lasting, speaking to the importance of interventions that can maintain affordability and access to these foods in creating sustainable changes.

### Limitations

Our study has several limitations. Our analysis sample is limited to applicants who responded to the follow-up survey 6 months after they were randomly assigned to enrollment or a waiting list, potentially limiting the generalizability of our results. We also used short screeners for outcome measures, including a 2-item screener to assess food insecurity, which prevents us from measuring degrees of food insecurity. Additionally, without retailer data, we lack data on specific purchases made by participants, limiting our information on consumption to self-reported intake.

## Conclusions

In this cohort study of a healthy food benefit program that fully subsidized $40 of fruit and vegetable purchases and allowed households to redeem benefits at grocery stores located near their home, participants randomly assigned to receive the benefit reported improved food security and an increased rate of eating fruits and vegetables 3 or more times per day, relative to those on a waiting list. Future research into similar programs should investigate whether those gains ultimately translate into health benefits for recipients.

## Notes

Supplement 1.
eMethods.
**eFigure 1.** Timeline and Description of Treatment Group Samples for Intervention Group 1 and 2 **eFigure 2.** Conceptual Model for Program Evaluation **eTable 1.** Summary Statistics by Treatment Group for Full Population of Applicants **eTable 2.** Association Between Program Applicant Characteristics, Treatment Assignment, and Response to Follow-Up Survey **eTable 3.** Missingness of Baseline Survey Responses for Study Sample **eTable 4.** Missingness of Follow-up Survey Responses for Study Sample **eTable 5.** Regression Coefficients for Analysis of Heterogeneous Effects of Treatment by Income Group **eTable 6.** Regression Coefficients for Analysis of Heterogeneous Effects of Treatment by Racial and Ethnic Group **eTable 7.** Regression Coefficients for Analysis of Heterogeneous Effects of Treatment by Preferred Survey Language **eTable 8.** Regression Coefficients for Analysis of Heterogeneous Effects of Treatment by Baseline Food Insecurity **eTable 9.** Average Effect for New Enrollment and Dropped Enrollment Among Those Without Missingness **eTable 10.** Separate Fruit and Vegetable Baseline and Endline Outcomes by Treatment Group **eTable 11.** Average Effect for Separate Fruits and Vegetables for New Enrollment and Dropped Enrollment
eReferences.

Supplement 2.
Data Sharing Statement
