# Supplementary material for: Healthy Food Benefit Programs, Fruit and Vegetable Consumption, and Food Security
Source: JAMA Netw Open. 2025 Aug 19;8(8):e2527601. doi: 10.1001/jamanetworkopen.2025.27601 (PMC12365700; doi:10.1001/jamanetworkopen.2025.27601)
Supplement: Supplement 1. — eMethods. eFigure 1. Timeline and Description of Treatment Group Samples for Intervention Group 1 and 2 eFigure 2. Conceptual Model for Program Evaluation eTable 1. Summary Statistics by Treatment Group for Full Population of Applicants eTable 2. Association Between Program Applicant Characteristics, Treatment Assignment, and Response to Follow-Up Survey eTable 3. Missingness of Baseline Survey Responses for Study Sample eTable 4. Missingness of Follow-up Survey Responses for Study Sample eTable 5. Regression Coefficients for Analysis of Heterogeneous Effects of Treatment by Income Group eTable 6. Regression Coefficients for Analysis of Heterogeneous Effects of Treatment by Racial and Ethnic Group eTable 7. Regression Coefficients for Analysis of Heterogeneous Effects of Treatment by Preferred Survey Language eTable 8. Regression Coefficients for Analysis of Heterogeneous Effects of Treatment by Baseline Food Insecurity eTable 9. Average Effect for New Enrollment and Dropped Enrollment Among Those Without Missingness eTable 10. Separate Fruit and Vegetable Baseline and Endline Outcomes by Treatment Group eTable 11. Average Effect for Separate Fruits and Vegetables for New Enrollment and Dropped Enrollment eReferences. [file jamanetwopen-e2527601-s001.pdf]

Supplemental Online Content

Knox MA, Wallace J, Baquero B, Hara-Hubbard K, Jones-Smith J. Randomized implementation of a healthy food benefit program. *JAMA Netw Open*. 2025;8(8):e2527601. doi:10.1001/jamanetworkopen.2025.27601

eMethods.
eFigure 1. Timeline and Description of Treatment Group Samples for Intervention Group 1 and 2
eFigure 2. Conceptual Model for Program Evaluation
eTable 1. Summary Statistics by Treatment Group for Full Population of Applicants
eTable 2. Association Between Program Applicant Characteristics, Treatment Assignment, and Response to Follow-Up Survey
eTable 3. Missingness of Baseline Survey Responses for Study Sample
eTable 4. Missingness of Follow-up Survey Responses for Study Sample
eTable 5. Regression Coefficients for Analysis of Heterogeneous Effects of Treatment by Income Group
eTable 6. Regression Coefficients for Analysis of Heterogeneous Effects of Treatment by Racial and Ethnic Group
eTable 7. Regression Coefficients for Analysis of Heterogeneous Effects of Treatment by Preferred Survey Language
eTable 8. Regression Coefficients for Analysis of Heterogeneous Effects of Treatment by Baseline Food Insecurity
eTable 9. Average Effect for New Enrollment and Dropped Enrollment Among Those Without Missingness
eTable 10. Separate Fruit and Vegetable Baseline and Endline Outcomes by Treatment Group
eTable 11. Average Effect for Separate Fruits and Vegetables for New Enrollment and Dropped Enrollment
eReferences.

This supplemental material has been provided by the authors to give readers additional information about their work.

1. **eMethods.**
    a. **Covariate Selection.**

While our treatments are randomly assigned which should result in balance of measured and unmeasured characteristics across treatment and comparison groups, we *a priori* chose to include variables that were predictive of the outcome to increase precision. Because several questions included many potential responses, we only included indicator variables for those categories that were chosen by 10% or more of respondents. Categories with responses under that threshold were classified as “Other”.

We divided our sample into a training and testing set, and used Least Absolute Shrinkage and Selection Operator (LASSO) with three different specifications to identify which covariates made the best predictions of each pre-treatment outcome in the testing set. To choose which covariates to include in our outcome models, we split our sample into training and testing samples, and used several methods, including linear regression, LASSO with cross validation, and elastic net, to find which covariates were predictive of the baseline outcomes. We find that the predictive power of our covariates is low across all methods, but that linear regression with all available covariates included has the best predictive performance in our test sample. Based on the LASSO results, we include the following covariates and categories in all primary models: Presence of children under 18 in household (yes/no), household income (greater than or less than 200% of federal poverty line for household size), race/ethnicity (Asian, Black/African American, White, Other, and Missing (includes Prefer not to answer, or Prefer to self-describe), age (above or below 60 years), preferred survey language (English, Chinese, Vietnamese, Other, and Missing (includes Prefer not to answer, or Prefer to self-describe)), preferred retailer accept Fresh Bucks (indicator variable for households naming a retailer that accepts Fresh Bucks). We include household size as a continuous variable. Finally, we included baseline levels of the outcomes of interest for each participant, also known as the ANCOVA specification.

    b. **Treatment Groups 1 & 2**

Treatment Group 1 is an analysis estimating the impact of gaining the Fresh Bucks benefit for 6 months, relative to an unenrolled comparison group. We use the October 2021 application survey to construct baseline measures of outcomes while all new applicants are untreated. We then use the July 2022 survey to construct our endline outcomes after either six months of program enrollment (treatment) or waitlist assignment (control).

Treatment Group 2 is an analysis estimating the impact of losing the Fresh Bucks benefit for 6 months after having been enrolled for a year or more, relative to a comparison group that stays

© 2025 Knox MA et al. *JAMA Network Open*.

continuously enrolled. We use the October 2021 application survey to construct baseline measures of outcomes while all returning applicants are still enrolled in the program. We then use the July 2022 survey to construct our endline outcomes after either six months of being off the program and on a waitlist (treatment) or continuous enrollment (control).

eFigure 1. Timeline and Description of Treatment Group Samples for Treatment Arm 1 and 2

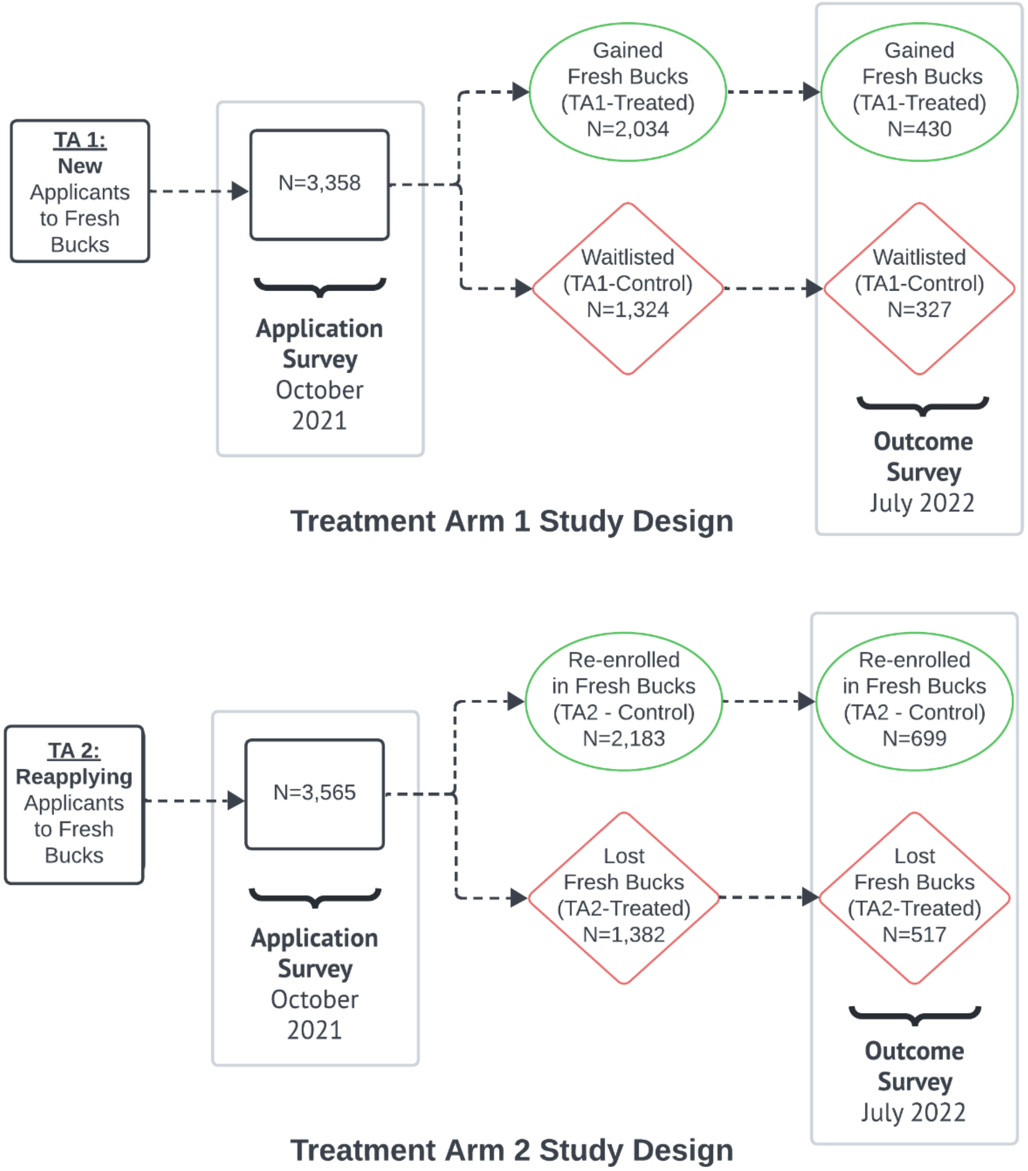

c. Conceptual Model

Because Seattle's Fresh Bucks program was designed to eliminate many of the systemic barriers to healthy food access that are unaddressed by other fruit and vegetable subsidy programs[1], we

© 2025 Knox MA et al. *JAMA Network Open*.

also focused our analysis on heterogeneity and equity in utilization by race, ethnicity, age, and geography. We examined whether Seattle's program design increases the equity of program implementation, a key requirement for a food policy to truly address the social determinants of health and health equity.

eFigure 2 displays the mechanisms by which we expect Fresh Bucks to result in improved dietary outcomes, food insecurity and cardiometabolic health outcomes. We expect that participation in the program will result in improved fruit and vegetable purchasing and intake. Additionally, evidence from previous nutrition incentive programs suggests that total diet quality may improve in response to fruit and vegetable incentive programs[2–4]. The substitution toward fruits and vegetables and away from unhealthy foods found in previous studies could impact both diet quality and energy balance, potentially reversing or slowing gains in BMI over time, and impacting biomarkers that are responsive to improved diet quality such as blood pressure and blood lipids[5,6]. Aside from the direct pathway of Fresh Bucks on biomarkers through promoting diet quality, we also hypothesize that the program could work to affect these outcomes through decreasing food insecurity and potentially freeing up a small amount of funds to be used for other needs[7,8]. We speculate that decreasing food insecurity would decrease stress, and that decreased stress could lead to improved diet quality since stress has been associated with high cortisol and increased propensity to consume high energy density foods[9].

eFigure 2. Conceptual Model for Program Evaluation

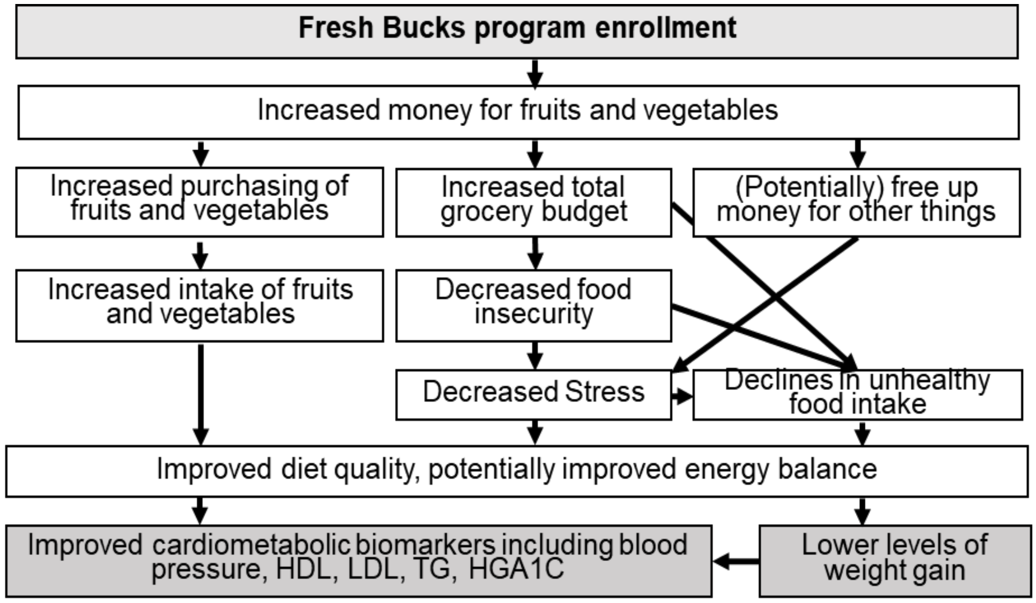

© 2025 Knox MA et al. *JAMA Network Open*.

2. Sample Selection, Missingness, and Internal Validity

Demographic characteristics for the full sample of applicants assigned to treatment via random draw (N=6,923) are given in Supplemental Table 1. These results show that treatment assignment was balanced across baseline demographic characteristics, validating our assumption that treatment was randomly assigned. Additionally, Tables 1 and 2 show within-treatment-group-balance across both covariates and pre-treatment outcomes in our analysis sample (among those who responded to the survey). These findings support our assumption that the treated and control analysis groups are similar in unobserved characteristics and that our treatment effect estimates are unbiased.

To better understand the generalizability of our findings, we examine which characteristics are associated with survey response and inclusion in the analysis sample by treatment group in Supplemental Table 2. For both treatment groups, survey respondents are more likely to be on the waitlist, speak Vietnamese, and be over 60. We additionally find that survey respondents in GA1 are more likely to have income >=200% of the federal poverty line, and less likely to be a race/ethnicity other than Asian, Black, or White than non-respondents. Respondents in GA2 are more likely to be White than non-respondents. Given the heterogeneities we find by race and ethnicity, these differences may limit the generalizability of our results to the full population of applicants.

The number and proportion of missing responses in baseline (pre-program) and follow-up (post program) survey data for our study sample are shown by question type in Supplemental Tables 3 and 4, respectively. Missing responses include both unanswered questions and "prefer not to say" responses. To account for these missing values without excluding these respondents from the study, we use an indicator variable for missing data for all variables. Additionally, we impute missing values of categorical variables with zero and impute missing values of continuous variables with the treatment group mean. We additionally include an indicator variable for missing values of each relevant variable. This procedure leads to consistent estimates of the treatment effect as long as missingness is not determined by treatment status.[10]

© 2025 Knox MA et al. *JAMA Network Open*.

eTable 1. Summary Statistics by Treatment Group for Full Population of Applicants

	Overall	Gained Fresh Bucks (Treatment Group 1)		Lost Fresh Bucks (Treatment Group 2)	
		Enrolled	Waitlisted	Dropped	Continuous
	N (%)	N (%)	N (%)	N (%)	N (%)
Sample Size	6923	2034	1324	1382	2183
Children in the Household					
No	4993 (72)	1419 (70)	926 (70)	1024 (74)	1624 (74)
Yes	1766 (26)	564 (28)	368 (28)	329 (24)	505 (23)
Prefer not to answer	164 (2.0)	51 (2.5)	30 (2.3)	29 (2.1)	54 (2.5)
Household Income Percent of Poverty Line					
<=200% FPL	5703 (82)	1614 (79)	1045 (79)	1154 (84)	1890 (87)
>200% FPL	1220 (18)	420 (21)	279 (21)	228 (17)	293 (13)
Household Size					
1	3522 (51)	979 (48)	644 (49)	734 (53)	1165 (53)
2	1694 (24)	487 (24)	317 (24)	339 (25)	551 (25)
3	698 (10)	233 (12)	165 (13)	117 (8.5)	183 (8.4)
4	542 (8.0)	173 (8.5)	103 (7.8)	101 (7.3)	165 (7.6)
5+	467 (7.0)	162 (8.0)	95 (7.2)	91 (6.6)	119 (5.5)
Race/Ethnicity					
Asian	2991 (43)	823 (41)	563 (43)	635 (46)	970 (44)
White	1653 (24)	446 (22)	298 (23)	358 (26)	551 (25)
Black	995 (14)	341 (17)	201 (15)	164 (12)	289 (13)
Another race/ethnicity	852 (12)	283 (14)	182 (14)	151 (11)	236 (11)
Missing	432 (6.0)	141 (6.9)	80 (6.0)	74 (5.4)	137 (6.3)
Preferred Language					
English	4207 (61)	1250 (62)	821 (62)	832 (60)	1304 (60)
Chinese	1594 (23)	419 (21)	264 (20)	345 (25)	566 (26)
Vietnamese	474 (7.0)	119 (5.9)	89 (6.7)	112 (8.1)	154 (7.1)
Another language	593 (9.0)	233 (12)	141 (11)	80 (5.8)	139 (6.4)
Missing	55 (1.0)	13 (0.64)	9 (0.68)	13 (0.94)	20 (0.92)
Age					
18-59 years old	3645 (53)	1249 (61)	833 (63)	618 (45)	945 (43)
60+ years old	3194 (46)	758 (37)	474 (36)	751 (54)	1211 (56)
Missing	84 (1.0)	27 (1.3)	17 (1.3)	13 (0.94)	27 (1.2)

Source/Notes: SOURCE: Authors' analyses of Fresh Bucks program data. NOTES: Respondent demographic characteristics based on survey responses from October 2021 (baseline) and June 2022 (endline). Tests of covariate balance between treatment and control groups within treatment groups were conducted by category using Chi-squared tests. All test statistics (not shown) were insignificant at the 10% level with the exception of income differences between treatment and control in treatment group 2. Baseline responses were collected for all lottery entrants (N=6,923) and endline responses (N=1,973) are from those who returned survey mailed to all lottery entrants 6-months post- lottery. Full baseline responses for analysis sample entrants found in Table 1.

© 2025 Knox MA et al. *JAMA Network Open*.

eTable 2. Association Between Program Applicant Characteristics, Treatment Assignment, and Response to Follow-Up Survey

	Gained Fresh Bucks (Treatment Group 1) Coefficient (SE)	Lost Fresh Bucks (Treatment Group 2) Coefficient (SE)
Treatment Assignment (Waitlist reference)		
Enrolled	-0.037*** (0.014)	0.055*** (0.016)
Child in household		
Yes	-0.011 (0.022)	-0.021 (0.028)
Missing Data about Children	-0.014 (0.048)	-0.021 (0.056)
FPL >200	0.042** (0.017)	-0.0063 (0.023)
Race/ethnicity (Asian reference)		
White	0.034 (0.024)	0.048* (0.027)
Black	-0.023 (0.025)	-0.0011 (0.031)
Another race/ethnicity	-0.049* (0.026)	-0.015 (0.032)
Missing	-0.016 (0.034)	-0.038 (0.039)
Language (English reference)		
Chinese	0.020 (0.024)	0.038 (0.027)
Vietnamese	0.096*** (0.034)	0.13*** (0.036)
Another language	-0.0032 (0.024)	-0.011 (0.033)
Missing	0.078 (0.090)	-0.064 (0.085)
Age		
>60 years old	0.21*** (0.017)	0.21*** (0.020)
Missing Age Data	0.0086 (0.067)	0.019 (0.080)
Normal HH size	-0.052 (0.060)	-0.0045 (0.077)
Observations	3358	3565

Source/Notes: SOURCE: Authors' analyses of Fresh Bucks program data. NOTES: *** p<.01, ** p<.05, * p<0.1. Robust standard errors are in parentheses.

© 2025 Knox MA et al. *JAMA Network Open*.

eTable 3. Missingness of Baseline Survey Responses for Study Sample

	Missing, N	Missing, %
Baseline Fruit	83	4.2
Baseline Green Veg	82	4.2
Baseline Potato	102	5.2
Baseline Fried Potato	103	5.2
Baseline Other Veg	86	4.4
Baseline Food Worried	256	13
Baseline Food Ran Out	306	16
Baseline Food Security (missing for either of above)	219	11
Children in HH	38	1.9
Household Income Category	0	0
Household Size	0	0
Race/Ethnicity	87	4.4
Age	14	0.71
Preferred Language	12	0.61

SOURCE: Authors' analyses of Fresh Bucks program data.

eTable 4. Missingness of Follow-up Survey Responses for Study Sample

	Missing, N	Missing, %
Follow-up Fruit	5	0.25
Follow-up Green Veg	6	0.30
Follow-up Potato	16	0.81
Follow-up Fried Potato	7	0.35
Follow-up Other Veg	10	0.51
Follow-up Food Worried	15	0.71
Follow-up Food Ran Out	11	0.56
Follow-up Food Security (missing for either of above)	20	1.0

SOURCE: Authors' analyses of Fresh Bucks program data.

© 2025 Knox MA et al. *JAMA Network Open*.

3. Regression Models

To estimate average treatment effects, we estimated the following linear equation

$$y_i = \beta_0 + \beta_1 T_i + X_i{}'\Gamma + \theta y_i^0 + \varepsilon_i$$

Where yi is one of our three outcomes of interest, Ti is an indicator for treatment assignment, yio is the value of yi reported by the individual in the baseline survey, and Xi are a vector of baseline demographic characteristics, as discussed in the manuscript. The estimate of the average treatment effect comes from the estimate of b1.

To estimate heterogeneities in the treatment effect by respondent characteristic, we estimate the following linear equation

$$y_i = \beta_0 + \beta_1 T_i + \beta_2 T_i \times Z_i + X_i{}'\Gamma + \theta y_i^0 + \varepsilon_i$$

Where the additional term $T_i \times Z_i$ is the interaction between treatment assignment and the categories for the demographic characteristic in question (e.g. indicator variables for each race category tested are interacted with treatment to assess heterogeneities by race). The treatment effect on each separate group, as shown in Figures 4 and 5, is calculated from $\beta_1 + \beta_2$. Confidence intervals are calculated using the lincom command in Stata 18.

To estimate the effect of treatment on outcome quantiles, rather than on the average outcome, we estimate the following set of β_τ coefficients, where t are the quantiles 10, 20,…90, by solving the following minimization problem

$$\beta_\tau = \ argmin_\beta \left[\tau \sum_{i:y_i > x_i\beta} |y_i - x_i\beta| \ + \ (1 - \tau) \sum_{i:y_i < x_i\beta} |y_i - x_i\beta| \right]$$

The resulting estimates give the amount by which the tth percentile of y is shifted for the treated group relative to the control group.

© 2025 Knox MA et al. *JAMA Network Open*.

4. **Results**
   a. **Raw Regression Results for Heterogeneity Analysis**

eTable 5. Regression Coefficients for Analysis of Heterogeneous Effects of Treatment by Income Group

	Gained Fresh Bucks (Treatment Group 1) Coefficient (SE)	Lost Fresh Bucks (Treatment Group 2) Coefficient (SE)
Treatment	0.37** (0.16)	-0.37*** (0.12)
Income >200% FPL	0.60** (0.26)	-0.13 (0.19)
Treatment x Income>200% FPL	-0.73** (0.32)	-0.0019 (0.29)
Constant	2.8 (0.26)	2.3 (0.28)
Observations	722	1183
F-test of joint significance	5.25**	0.00

Source/Notes: SOURCE: Authors' analyses of Fresh Bucks program data. NOTES: *** p<.01, ** p<.05, * p<0.1. Robust standard errors are in parentheses. Regression results exclude those with missing F/V consumption data at baseline or follow-up.

eTable 6. Regression Coefficients for Analysis of Heterogeneous Effects of Treatment by Racial and Ethnic Group

	Gained Fresh Bucks (Treatment Group 1) Coefficient (SE)	Lost Fresh Bucks (Treatment Group 2) Coefficient (SE)
Treatment	-0.10 (0.22)	-0.32* (0.16)
White	-1.1*** (0.28)	-0.49** (0.20)
Black	-1.2*** (0.36)	-0.37 (0.26)
Another race/ethnicity	-0.58 (0.51)	-0.19 (0.32)
Missing	-1.9*** (0.41)	0.36 (0.45)
Treatment x White	0.55* (0.30)	0.068 (0.23)
Treatment x Black	0.91** (0.45)	-0.18 (0.39)
Treatment x Another race/ethnicity	0.021 (0.58)	-0.48 (0.37)
Treatment x Missing	1.5*** (0.50)	-0.50 (0.67)
Constant	3.0 (0.28)	2.3 (0.28)
Observations	722	1183
F-test of joint significance	2.89**	0.71

Source/Notes: SOURCE: Authors' analyses of Fresh Bucks program data. NOTES: *** p<.01, ** p<.05, * p<0.1. Robust standard errors in parentheses. Regression results exclude those with missing F/V consumption data at baseline or follow-up.

© 2025 Knox MA et al. *JAMA Network Open*.

eTable 7. Regression Coefficients for Analysis of Heterogeneous Effects of Treatment by Preferred Survey Language

	Gained Fresh Bucks (Treatment Group 1) Coefficient (SE)	Lost Fresh Bucks (Treatment Group 2) Coefficient (SE)
Treatment	0.37** (0.17)	-0.55*** (0.13)
Chinese	-0.79*** (0.22)	-0.49*** (0.18)
Vietnamese	-0.70** (0.28)	-0.49** (0.23)
Another language	-0.58* (0.32)	-0.44* (0.24)
Missing	-1.1*** (0.31)	0.10 (0.36)
Treatment x Chinese	-0.21 (0.34)	0.33 (0.25)
Treatment x Vietnamese	-1.3** (0.56)	0.28 (0.41)
Treatment x Another language	0.36 (0.52)	1.0* (0.56)
Treatment x Missing	-1.1** (0.54)	-1.7* (0.91)
Constant	2.8 (0.27)	2.4 (0.28)
Observations	722	1183
F-test of joint significance	2.56**	2.31*

Source/Notes: SOURCE: Authors' analyses of Fresh Bucks program data. NOTES: *** p<.01, ** p<.05, * p<0.1. Robust standard errors are in parentheses. Regression results exclude those with missing F/V consumption data at baseline or follow-up.

eTable 8. Regression Coefficients for Analysis of Heterogeneous Effects of Treatment by Baseline Food Insecurity

	Gained Fresh Bucks (Treatment Group 1) Coefficient (SE)	Lost Fresh Bucks (Treatment Group 2) Coefficient (SE)
Treatment	0.14 (0.17)	-0.38*** (0.13)
Food Secure	0.0082 (0.26)	-0.0067 (0.17)
Missing	-0.22 (0.26)	0.087 (0.21)
Treatment x Food Secure	0.083 (0.34)	0.055 (0.25)
Treatment x Missing	0.40 (0.38)	-0.13 (0.33)
Constant	3.3 (0.26)	2.9 (0.28)
Observations	756	1216
F-test of joint significance	0.55	0.13

Source/Notes: SOURCE: Authors' analyses of Fresh Bucks program data. NOTES: *** p<.01, ** p<.05, * p<0.1. Robust standard errors are in parentheses. Regression results exclude those with missing F/V consumption data at baseline or follow-up.

© 2025 Knox MA et al. *JAMA Network Open*.

b. Sensitivity and Secondary Analyses

eTable 9. Average Effect for New Enrollment and Dropped Enrollment Among Those Without Missingness

	Gained Fresh Bucks (Treatment Group 1) Coefficient (SE)	Lost Fresh Bucks (Treatment Group 2) Coefficient (SE)
Food Security Prevalence	7.5** (3.0)	-3.1 (2.4)
Observations	582	962
Continuous F/V Consumption	0.20 (0.15)	-0.36*** (0.11)
Observations	640	1068
F/V Consumption 3x/day Prevalence	6.1 (3.9)	-6.5** (3.0)
Observations	640	1068

Source/Notes: SOURCE: Authors' analyses of Fresh Bucks program data. NOTES: *** p<.01, ** p<.05, * p<0.1. Robust standard errors are in parentheses.

© 2025 Knox MA et al. *JAMA Network Open*.

eTable 10. Separate Fruit and Vegetable Baseline and Endline Outcomes by Treatment Group

	Gained Fresh Bucks (Treatment Group 1)		Lost Fresh Bucks (Treatment Group 2)	
	Enrolled	Waitlisted	Dropped	Continuous
Continuous Fruit times per Day, Mean (sd)				
Baseline	0.58 (0.55)	0.57 (0.56)	0.73 (0.59)	0.74 (0.61)
Endline	1.1 (0.68)	0.90 (0.68)	0.92 (0.69)	1.1 (0.67)
Observations	403	311	493	678
Continuous Vegetable times per Day, Mean (sd)				
Baseline	1.4 (1.3)	1.4 (1.3)	1.6 (1.2)	1.6 (1.2)
Endline	2.0 (1.3)	2.0 (1.5)	1.9 (1.4)	2.1 (1.4)
Observations	408	314	496	684

Source/Notes: SOURCE: Authors' analyses of Fresh Bucks program data. NOTES: *** p<.01, ** p<.05, * p<0.1. Robust standard errors are in parentheses. Results exclude those with missing fruit or vegetable consumption data, respectively, at baseline or follow-up.

eTable 11. Average Effect for Separate Fruits and Vegetables for New Enrollment and Dropped Enrollment

	Gained Fresh Bucks (Treatment Group 1) Coefficient (SE)	Lost Fresh Bucks (Treatment Group 2) Coefficient (SE)
Continuous Fruit Consumption	0.20*** (0.051)	-0.15*** (0.039)
Observations	714	1171
Continuous Vegetable Consumption	0.020 (0.10)	-0.21*** (0.080)
Observations	722	1180

Source/Notes: SOURCE: Authors' analyses of Fresh Bucks program data. NOTES: *** p<.01, ** p<.05, * p<0.1. Robust standard errors are in parentheses. Regression results exclude those with missing fruit or vegetable consumption data, respectively, at baseline or follow-up.

© 2025 Knox MA et al. *JAMA Network Open*.

eReferences

1. Freedman DA, Blake CE, Liese AD. Developing a Multicomponent Model of Nutritious Food Access and Related Implications for Community and Policy Practice. *Journal of Community Practice*. 2013;21(4):379-409. doi:10.1080/10705422.2013.842197

2. Alaofè H, Freed N, Jones K, Plano A, Taren D. Impacts of Double Up SNAP Farmers' Market Incentive Program on Fruit and Vegetable Access, Purchase and Consumption. *Journal of Nutrition and Health Sciences*. 2017;4(3). doi:10.15744/2393-9060.4.304

3. Lowery CM, Henderson R, Curran N, Hoeffler S, Marco MD, Ng SW. Was a short-term COVID-relief fruit and vegetable subsidy program associated with changes in grocery purchases? How did Healthy Helping participants' grocery purchases change during the program ? In: *Healthy Eating Research*. ; 2021:2021.

4. Collins AM, Klerman JA. Improving Nutrition by Increasing Supplemental Nutrition Assistance Program Benefits. *American Journal of Preventive Medicine*. 2017;52(2):S179-S185. doi:10.1016/j.amepre.2016.08.032

5. Jenkins DJA, Popovich DG, Kendall CWC, et al. Effect of a diet high in vegetables, fruit, and nuts on serum lipids. *Metabolism: Clinical and Experimental*. 1997;46(5):530-537. doi:10.1016/S0026-0495(97)90190-6

6. John JH, Ziebland S, Yudkin P, Roe LS, Neil HAW. Effects of fruit and vegetable consumption on plasma antioxidant concentrations and blood pressure: A randomised controlled trial. *Lancet*. 2002;359(9322):1969-1974. doi:10.1016/S0140-6736(02)98858-6

7. Cook M, Ward R, Newman T, et al. Food Security and Clinical Outcomes of the 2017 Georgia Fruit and Vegetable Prescription Program. *Journal of Nutrition Education and Behavior*. 2021;53(9):770-778. doi:10.1016/j.jneb.2021.06.010

8. Byker Shanks C, Uy WF, Zhang N, et al. Nutrition Incentives Associated With Improved Outcomes: 2020–2023 Results From the U.S Gus Schumacher Nutrition Incentive Program. *AJPM Focus*. 2025;4(4):100348. doi:10.1016/j.focus.2025.100348

9. Dallman MF. Stress-induced obesity and the emotional nervous system. *Trends in Endocrinology and Metabolism*. 2010;21(3):159-165. doi:10.1016/j.tem.2009.10.004

10. Zhao A, Ding P. To Adjust or not to Adjust? Estimating the Average Treatment Effect in Randomized Experiments with Missing Covariates. *Journal of the American Statistical Association*. 2022;0(0):1-11. doi:10.1080/01621459.2022.2123814

© 2025 Knox MA et al. *JAMA Network Open*.
