# Supplementary material for: Healthy Food Benefit Programs, Fruit and Vegetable Consumption, and Food Security
Source: JAMA Netw Open. 2025 Aug 19;8(8):e2527601. doi: 10.1001/jamanetworkopen.2025.27601 (PMC12365700; doi:10.1001/jamanetworkopen.2025.27601)
Supplement: Supplement 2. — Data Sharing Statement [file jamanetwopen-e2527601-s002.pdf]

Data Sharing Statement

Knox. Randomized Implementation of a Healthy Food Benefit Program. *JAMA Netw Open*. Published August 19, 2025. doi:10.1001/jamanetworkopen.2025.27601

Data
Data available: Yes
Data types: Data dictionary
How to access data: We will make data dictionary available upon publication at osf.io. Access to de-identified study data may be available from knoxm@uw.edu with permission from the City of Seattle.
When available: With publication

Supporting Documents
Document types: Statistical/analytic code
How to access documents: Statistical code will be posted on osf.io.
When available: With publication

Additional Information
Who can access the data: Anyone requesting the data.
Types of analyses: Any purpose.
Mechanisms of data availability: The requestor may need a signed data use agreement with the City of Seattle.
